# Enhanced Bioactivity of *Puerariae* Radix‐ *Hovenia* Seed Extracts Through *Lactiplantibacillus plantarum* and *Lacticaseibacillus paracasei* Co‐Fermentation: Impact on Alcoholic Liver Injury and Macrophage Polarization

**DOI:** 10.1002/fsn3.71829

**Published:** 2026-04-25

**Authors:** Yunpeng Sun, Shen Yao, Yuan Cui, Yiyu Ni, Meichen Liu, Daqing Zhao, Shiting Yu, Siming Wang

**Affiliations:** ^1^ Northeast Asia Research Institute of Traditional Chinese Medicine Changchun University of Chinese Medicine Changchun China; ^2^ College of Traditional Chinese Medicine Jilin Agricultural Science and Technology College Jilin China

**Keywords:** alcoholic liver injury, co‐fermentation, *Hovenia* seed, inflammatory, *Puerariae* Radix

## Abstract

This study investigated the hepatoprotective potential of *Puerariae* Radix‐*Hovenia* Seed extracts co‐fermented with *Lactiplantibacillus plantarum* and *Lacticaseibacillus paracasei* in alleviating alcoholic liver injury (ALI) and regulating macrophage polarization. Network pharmacology analysis identified 29 core overlapping targets between bioactive components and ALI‐related targets. Co‐fermentation elevated total flavonoids, polysaccharides, and saponins, and altered the flavonoid profile: puerarin (+20%), daidzin (+695%), and myricetin (+84%) increased, while daidzein decreased by 48%. Optimized structural degradation and reduced impurity fragments indicated advantages in enhancing bioavailability and reducing adverse reactions. Co‐fermented extract (PHF) ameliorated alcohol intoxication, activated alcohol‐metabolizing enzymes, reduced blood ethanol and acetaldehyde levels; mitigated hepatotoxicity, hepatosteatosis, and fibrosis. PHF exhibited dual immunomodulatory effects via TLR4/MyD88/NF‐κB pathway by balancing inflammatory cytokines and regulating macrophage polarization. Its natural origin and dual immune properties boost translational potential for ALI.

## Graphical abstract

*Puerariae* Radix and *Hovenia* Seed were co‐fermented with *Lactiplantibacillus plantarum* and *Lacticaseibacillus paracasei* to produce PHF. In a mouse model of alcohol‐induced liver injury, PHF intervention alleviated liver damage. The underlying mechanism may be related to the regulation of macrophage polarization. PHF inhibited the pro‐inflammatory M1 polarization of macrophages by suppressing the TLR4/MyD88/NF‐κB signaling pathway and reducing the expression of iNOS, TNF‐α, IL‐1β, and CD80. Meanwhile, PHF promoted anti‐inflammatory M2 polarization by upregulating the IL‐10/STAT3 pathway and increasing Arg1 and CD206 expression, thereby exerting protective effects against alcoholic liver injury.

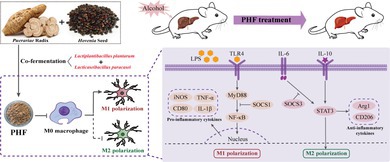

## 1 Introduction

In accordance with the Global Status Report on Alcohol and Health, approximately 3.3 million people die form alcohol‐related causes each year (Singal et al. 2018). Since the liver metabolizes over 80% of the alcohol, ALI is one of the most prevalent adverse consequence of excessive alcohol consumption. Persistent excessive alcohol intake can lead to a spectrum of liver pathologies, including hepatosteatosis, alcoholic hepatitis, liver fibrosis, and ultimately hepatocellular carcinoma, which are associated with high morbidity and mortality (Bajaj 2019; Kong et al. 2019; Mackowiak et al. 2024; Wu et al. 2023). Although glucocorticoids are frequently employed to address ALI, their application is limited by significant adverse effects (Pronko et al. 2002). Therefore, identifying natural products with fewer side effects for the prevention and adjuvant treatment of alcohol‐induced liver injury is of great importance. A major focus of current research is to elucidate how alcohol and its metabolites directly damage hepatocytes and induce inflammation, including neutrophil infiltration. In ALI and other inflammatory diseases, pathogenic stimuli trigger the release of pro‐inflammatory mediators that activate the immune system (Dou et al. 2019). Inflammatory response process is initiated when pathogen‐ or damage‐associated molecular patterns engage Toll‐like receptors (TLRs), subsequently activating intracellular signaling cascades via downstream proinflammatory transcription factors such as nuclear factor‐κB (NF‐κB) (Ciesielska et al. 2021; Liu et al. 2022; Mandrekar and Szabo 2009). Concurrently, macrophages undergo polarization in response to various stimuli including lipopolysaccharide (LPS) and tumor necrosis factor‐α (TNF‐α), differentiating into distinct functional phenotypes: the M1 subtype that amplifies inflammatory responses through proinflammatory cytokine production, and the M2 subtype that facilitates tissue repair (Mendes and Schnabl 2020; Van den Bossche et al. 2017). Current therapeutic strategies for ALI primarily focus on modulating these inflammatory pathways and immune responses, representing a targeted approach for both disease prevention and clinical management.

As a classic combination in Traditional Chinese Medicine (TCM) for managing alcohol‐related disorders, the herb pair of *Puerariae* Radix (PR) and *Hovenia* Seed (HS) has gained extensive application in the functional food sector in recent years. PR, a member of the Leguminosae family, is traditionally used for alcohol detoxification, as documented in the *Pharmacopoeia of the People's Republic of China*, was first described in “Shennong Bencao Jing” (Shennong's Classic of Materia Medica) and is now also used utilized as a functional food ingredient (Read 1930; Wong et al. 2011; Zhang et al. 2013). HS, a traditional medicinal plant documented in the “Bencao Gangmu” (Compendium of Materia Medica), has been historically used in East Asia for hepatoprotection and alcohol detoxification. The PR‐HS combination is pharmacologically rationalized in TCM due to their complementary actions. In our previous study, compared with the individual decoctions, the combined decoction of PR‐HS exhibited a more potent effect on ameliorating alcohol intoxication, particularly in delaying alcohol‐induced loss of righting reflex (LORR) and accelerating the recovery intoxication time. PR enhances gastrointestinal motility and accelerates ethanol metabolism, while HS diuretic and laxative effects, directly neutralizing alcohol‐derived toxins (Hyun et al. 2010; Wu et al. 2024; Zhang, Song, et al. 2024; Zhang, Yu, et al. 2024). The dual‐component regimen demonstrated significantly greater therapeutic efficacy than either component applied individually, as evidenced by synergistic reductions in hepatic transaminases, inflammatory markers, along with enhanced alcohol dehydrogenase (ADH) activity. These results are consistent with our preliminary experimental data (Chen, Zhang, et al. 2022; Lee et al. 2024; Paik et al. 2024). Notably, bioactive flavonoids in PR and HS, such as puerarin, daidzin, myricetin, and daidzein have been shown to ameliorate alcohol withdrawal symptoms and attenuate hepatic inflammation in rats, concurrently reducing transaminase and pro‐inflammatory cytokine levels. These collective findings suggest their potential therapeutic value against ALI (Chen et al. 2013; He et al. 2022; Hyun et al. 2010; Xiang et al. 2012).

Fermentation is a widely utilized processing technology in both the food and TCM industries, notably for enhancing the bioavailability and activity of bioactive compounds such as polyphenols, flavonoids, and saponins. This biotransformation process involves the cleavage of glycosidic bonds and the production of enzymes such as lactase, thereby promoting the formation of beneficial microbial communities capable of synthesizing copious bioactive secondary metabolites (Ai et al. 2019). These changes collectively improve the release, solubility, and potential absorption of bioactive constituents in the gastrointestinal tract. Furthermore, the choice of fermentative strains is crucial, as different strains or their combinations can yield distinct and often synergistic effects on the overall bioactivity of the final product. Among the currently widely applied functional fermentative strains, *Lactiplantibacillus plantarum* (*Lp. plantarum*) can enhance the antioxidant activity of plant‐derived functional foods, *Lacticaseibacillus paracasei* strengthens the intestinal regulatory function of products, and *Saccharomyces cerevisiae* improves the digestibility of plant proteins while shortening the fermentation cycle (Junior et al. 2020; Vachkova et al. 2023; Wang et al. 2020; Zhou et al. 2025). Fermentation increased the content of isoflavones and phenolic acids in PR, resulting in a nearly 26% increase in puerarin content and improved anti‐inflammatory and antioxidant damage effects (Choi et al. 2020; Zhang et al. 2017). After fermentation with *Lp. plantarum*, the γ‐aminobutyric acid content in HS is increased, accompanied by a reduction in glutamate levels, thereby enhancing its hepatoprotective efficacy against ALI in mice (Park et al. 2020, 2023). Thus, fermentation enhances the therapeutic efficacy of functional foods by regulating the composition of bioactive substances, while concurrently reducing their adverse reactions and side effects.

This study employed HPLC‐MS analysis to compare the flavonoid profiles of fermented and nonfermented extracts from PR‐HS berb pair (designated PHF and NFE, respectively). The hepatoprotective effects of PHF against ALI and its underlying mechanisms were investigated using an in vivo ALI murine model and an in vitro LPS‐stimulated M1‐polarized RAW264.7 macrophage model. The findings provide a theoretical foundation for developing the PR‐HS combination, particularly in its fermented form, as a potential therapeutic agent for ALI.

## 2 Materials and Methods

### 2.1 Network Pharmacology Prediction

ALI‐associated targets were identified via CTD, GeneCards, OpenTargets, and HREB databases. After deduplication and data integration, 795 unique disease targets were compiled. Potential targets of PR‐HS were retrieved from TCMSP, BATMAN‐TCM, ETCM, and TCM‐ID databases, yielding 1768 candidate therapeutic targets. Venn diagram analysis (Draw Venn software) identified 239 overlapping targets between PR‐HS constituents and ALI pathogenesis. These common targets were subjected to STRING database for protein–protein interaction (PPI) network construction, with subsequent topo logical analysis performed in Cytoscape 3.9.1 to determine core targets. Constituent prioritization was executed using pharmacokinetic criteria (oral bioavailability ≥ 10%, drug‐likeness ≥ 0.18), followed by phyto chemical classification into four categories: flavonoids, lipids, triterpenoids, and other compounds.

### 2.2 Preparation of NFE and PHF

PR and HS were purchased from Hebei Province, China, and identified by Prof. Shichao Liu (Changchun University of Chinese Medicine, China). *Lp. plantarum*, *L. paracasei* , and *S. cerevisiae* , obtained from the China Conservation Centre for General Microbial Strains, were aerobically cultured in MRS solid medium (Solarbio, China) at 28°C with shaking at 180 rpm for 24 h. This culture was subsequently subcultured for three consecutive generations under identical conditions before use. Well‐grown single colonies were selected, and bacterial suspensions were mixed at a 1:1 ratio (Jung et al. 2019). Dried PR and HS were powdered, passed through an 80 mesh sieve, and mixed in equal proportions (by weight). The herbal mixture was then combined with distilled water at a solid‐to‐liquid ratio of 1:4 (g:mL), and the pH was adjusted to 7.0. The slurry was sterilized by autoclaving at 121°C for 15 min and cooled under UV light for 30 min. The mixed bacterial suspension was inoculated into the sterile medium at 2% (v/w, relative to the herbal mass). Fermentation was carried out on a shaker at 37°C, 180 rpm for 36 h (Yan et al. 2019). The dried material was then subjected to reflux extraction with six volumes of 60% ethanol (v/w) at 80°C for 1 h. After extraction, the mixture was filtered to remove insoluble substances, and the ethanol was evaporated under reduced pressure to obtain PHF. NFE was prepared following the same procedure but omitting the fermentation step.

### 2.3 HPLC‐Q‐TOF‐MS Analysis

Chromatographic separation was performed on an Acquity UPLC HSS T3 column (2.1 mm × 100 mm, 1.8 μm; Waters, USA) maintained at 30°C. The mobile phase consisted of (A) 0.1% formic acid in water and (B) methanol at a flow rate of 0.4 mL/min. A gradient elution program was applied as follows: 0–15 min, 70% A; 15–25 min, 70%–45% A; 25–35 min, 45% A; 35–45 min, 45%–70% A for column re‐equilibration. The injection volume was 5 μL.

Mass spectrometry detection was conducted using a quadrupole‐time‐of‐flight (Q‐TOF) mass spectrometer (Agilent 6546) equipped with an electrospray ionization (ESI) source operated in positive ion mode. The ion source parameters were optimized as follows: ion source temperature, 320°C; drying gas (N_2_) flow, 10 L/min; nebulizer gas pressure, 55 psi; capillary voltage, 3.5 kV; fragmentor voltage, 60 V. Data were acquired in full‐scan mode (*m*/*z* 100–1500) with auto MS/MS fragmentation (collision energy: 10–35 eV). All data were processed using MassHunter Qualitative Analysis 10.0 (Agilent) software for peak alignment, molecular feature extraction, and compound identification by matching against the Agilent TCM library (version V20‐04‐17).

### 2.4 Scanning Electron Microscopy Inspection

The samples were affixed to an aluminum column using double‐sided tape. The microstructures of the samples were examined at magnifications of 150×, 500×, and 1500× utilizing a field emission scanning electron microscope (Hitachi‐SU8010, Hitachi, Tokyo, Japan).

### 2.5 HPLC Analysis

Puerarin (CAS 3681‐99‐0, ≥ 98%), daidzin (CAS 552‐66‐9, ≥ 98%), daidzein (CAS 486‐66‐8, ≥ 98%), and myricetin (CAS 529‐44‐2, ≥ 98%), sourced from Yuanye Bio‐Technology (China), were dissolved in methanol to prepare a mixed standard solution at a concentration of 0.2 mg/mL. The fermented botanical drug extracts were dissolved in methanol. The chromatographic column was the ZORBAX Eclipse Plus C18 (4.6 × 250 mm 5‐Micron, Agilent, USA). The mobile phase (water, A; methanol, B) was implemented at a flow rate of 1.0 mL/min. A gradient elution program: 0–15 min, 70% A; 15–25 min, 70%–45% A; 25–35 min, 45% A; 35–45 min, 45%–70% A. The column temperature was maintained at 30°C and detected at 250 nm. The injection volume was 10 μL.

### 2.6 Animal Experiments

Animal experiments were approved by the Institutional Animal Ethics Committee of Changchun University of Chinese Medicine (Approval No. 2024281) and conducted in accordance with the ARRIVE guidelines. Seventy specific pathogen‐free (SPF) male ICR mice (4 weeks old, 20 ± 2 g; License No. SCXK‐2020‐0002) were obtained from Changchun Yisi, China. After 1 week of acclimatization, the mice were randomly assigned to experimental groups. To establish the ALI model, mice were gavaged with Chinese liquor (56% v/v alcohol) at a dose of 0.8 mL/kg body weight daily for eight consecutive days (Chen, Zhang, et al. 2022). Thirty minutes prior to each ethanol administration on these days, mice in the treatment groups received one of the following via gavage: Puerarin Schisandra tablets (positive, 50 mg/kg body weight/day), PHF (83, 166, or 249 mg/kg body weight/day), or the nonfermented extract (NFE, 166 mg/kg body weight/day), which was prepared identically to PHF but without the fermentation step. On the ninth day, following a 12‐h fasting period (with free access to water), the mice were fully anesthetized by an intraperitoneal injection of 1% sodium pentobarbital (50 mg/kg body weight). The depth of anesthesia was confirmed by the absence of a pedal withdrawal reflex upon toe pinching. Subsequently, euthanasia was performed via cervical dislocation, and death was verified by the cessation of respiration and heartbeat, along with pupil dilation. Their blood and intact liver were promptly collected and stored at −80°C for measurements of biochemical parameters (Lee 2004; Yuan et al. 2024; Zhang et al. 2009).

### 2.7 Histopathological Analyses

For hematoxylin and eosin (H&E) staining and Sirius red staining, liver tissues were placed in pre‐cooled petri dishes, rinsed with saline to remove blood residues, and immersed in 4% paraformaldehyde fixative for 24 h. Subsequently, the tissues were embedded in paraffin wax and sectioned into approximately 4‐μm‐thick slices. After dewaxing and rehydration, the sections were to be stained with either Sirius red staining medium or hematoxylin and eosin solution (Solarbio, China). The stained samples were examined under a light microscope, and images were captured and analyzed (Feng et al. 2019).

### 2.8 Western Blot (WB) Analysis

Total protein was extracted from liver tissues and cell suspensions using RIPA Lysis Buffer, and the concentration was quantified with a BCA Protein Assay Kit (Beyotime, Shanghai, China). Equal amounts of protein were then separated by 10% SDS‐PAGE and subsequently transferred onto nitrocellulose membranes. The membranes were blocked and then incubated overnight at 4°C with the following primary antibodies: β‐Actin, TLR4, MyD88, NF‐κB, alcohol dehydrogenase 1 (ADH1), acetaldehyde dehydrogenase 2 (ALDH2), glyceraldehyde‐3‐phosphate dehydrogenase (GAPDH), signal transducer and activator of transcription 3 (STAT3), suppressor of cytokine signaling 3 (SOCS3), inducible nitric oxide synthase (iNOS), interleukin‐10 (IL‐10), and arginase‐1 (Arg1) (Beyotime, China). After washing, the membranes were incubated with anti‐rabbit IgG antibody (Beyotime, China) for 2 h in the light at RT. Protein bands were visualized using chemiluminescence detection reagents and imaged with an iBright 1500 Imaging System (Thermo Fisher Scientific). Band intensities were quantified using ImageJ software (National Institutes of Health, USA).

### 2.9 Quantitative Reverse Transcription Polymerase Chain Reaction (qRT‐PCR) Analysis

Liver tissue was homogenized in TRIzol reagent, and total RNA was extracted using the RNAsimple Total RNA Kit (Tiangen, China). RNA concentration was measured using a DS‐11 FX+ spectrophotometer (DeNovix, USA). The extracted RNA was then reverse‐transcribed into cDNA using the TransScript One‐Step gDNA Removal and cDNA Synthesis SuperMix (TransGen, China). Primer sequences (designed by Primer Premier 6.0) are listed in Table S1. The qRT‐PCR was performed on a CFX Connect Real‐Time System (Bio‐Rad, USA). Gere expression levels were normalized to *β‐actin* (*Actb*) and calculated using the comparative 2^−ΔΔCT^ method.

### 2.10 Immunohistochemical Analysis

The liver tissue was fixed, paraffin‐embedded, and sectioned into 4‐μm pieces. The sections were deparaffinized and dehydrated in xylene and rehydrated through a graded ethanol series. After antigen retrieval and blocking with normal goat serum, the sections were incubated overnight at 4°C with the following primary antibodies: IL‐10, SOCS1, SOCS3, TLR4, MyD88, and NF‐κB p65 (Beyotime, USA). Subsequently, the sections were incubated with anti‐rabbit IgG secondary antibody (Beyotime, China). Color development was performed using a 3,3‐diaminobenzidine (DAB) substrate kit, resulting in brown‐yellow positive signals. Finally, the sections were counterstained with hematoxylin (blue nuclei), dehydrated, cleared, and mounted. Stained sections were examined and imaged under a Nikon E100 light microscope.

### 2.11 RAW264.7 Macrophage Cell Culture

RAW264.7 macrophage cells (RMCs, Procell, China) were seeded in 6‐well culture plates at a density of 1 × 10^5^ cells per well and cultured with dulbecco's modified eagle medium containing 10% fetal bovine serum and 1% penicillin for 24 h (Wu et al. 2018). The culture media was then replaced with LPS (LPS group, 0.5 μg/mL), IL‐4 (Positive group, 10 μg/mL), NFE (0.5 mg/mL), or PHF (0.1, 0.5, 1.0 mg/mL) to incubate RMCs for another 24 h. The RMCs incubated without LPS were used as a control (Normal group). 3‐(4,5‐dimethylthiazol‐2‐yl)‐2,5‐diphenyltetrazolium bromide (MTT) assays were performed to evaluate cell viability. After incubation, cells were incubated with 20 μL MTT solution (5 mg/mL in PBS) per well for 5 h. The formazan crystals were solubilized with 200 μL DMSO, and absorbance was measured at 570 nm using a microplate reader (Infinite 200 Pro, TECAN, Switzerland).

### 2.12 Flow Cytometry

RMCs were plated in 6‐well plates at 2 × 10^5^ cells/well. After the indicated treatments for 24 h, the cells were stained with CD80‐antibody, CD206‐antibody (Elabscience, China) for 30 min, and then incubated with macrophage lysates in the dark for 10 min before centrifuged at 328.7 × *g* for 5 min (4°C). Each tube was resuspended with PBS after the supernatants were removed. Ultimately, a flow cytometer was employed to conduct the flow cytometry analysis (Sartorius, Argentina, German) following standard settings (excitation light 488and 642 nm) (Njoroge et al. 2018).

### 2.13 Statistical Analysis

Results are presented as means ± standard deviation (SD). Comparisons between groups were performed using an Unpaired Student's *t*‐test (between two groups) or One‐way analysis of variance (ANOVA) followed by the Dunnett's post hoc test for comparisons with the Alcohol group. Statistical analyses were performed with GraphPadPrism 10.0 (GraphPad Software, USA).

## 3 Results

### 3.1 Network Pharmacology Analysis of PR‐HS in Treating ALI

**FIGURE 1:**
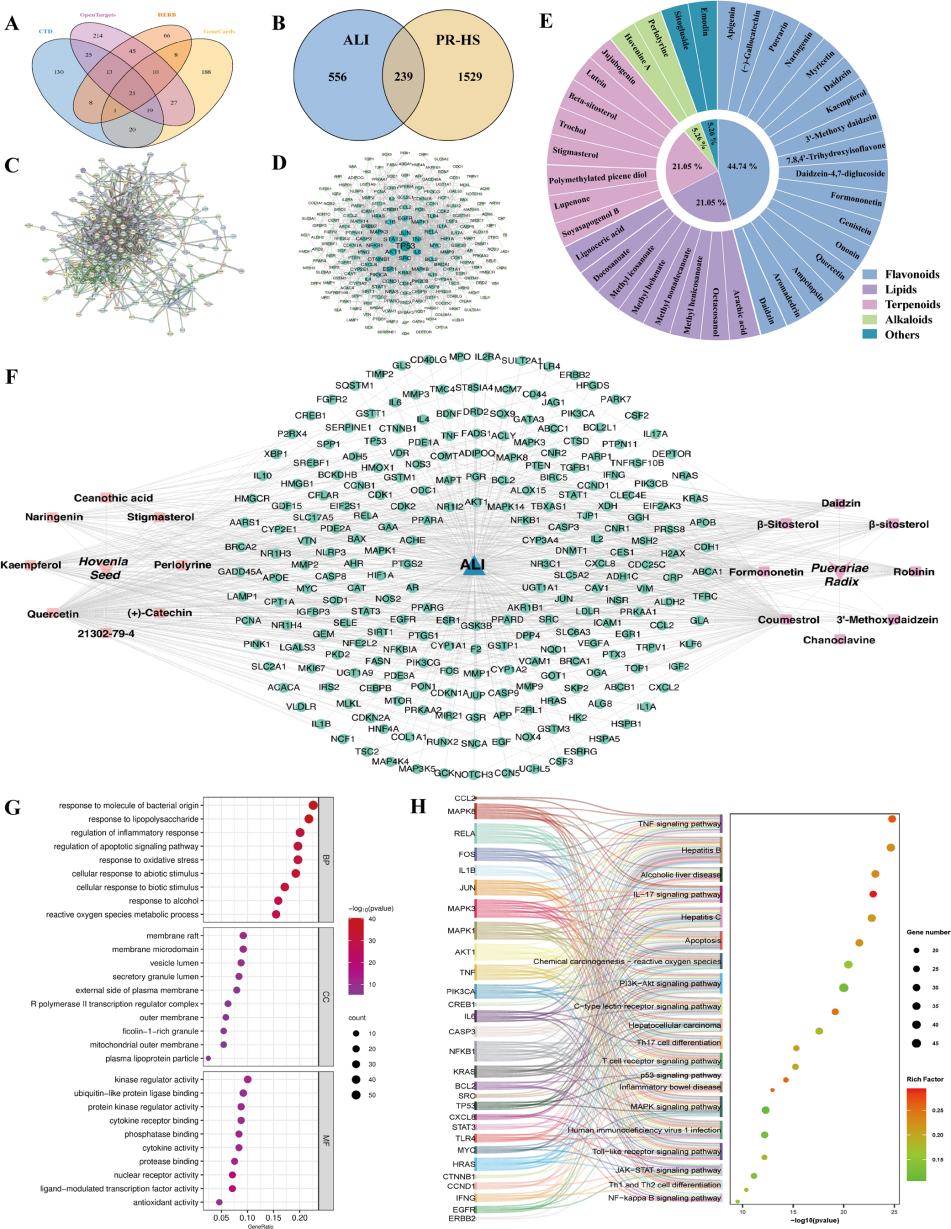
Pharmacological analysis of *Puerariae* Radix (PR)‐*Hovenia* See*d* (HS) with alcoholic liver injury (ALI) network. (A, B) Venn diagram demonstrating intersection targets of ALI in different databases, and intersection targets between PR‐HS bioactive components and ALI targets; (C) Protein–protein interaction network of potential therapeutic targets; (D) Identification of key therapeutic targets through topological analysis; (E) Phytochemical classification and bioactive constituent screening; (F) Drug‐compound‐target‐disease network diagram; (G, H) Gene ontology and KEGG analyses enrichment of potential therapeutic targets.

As illustrated in Figure 1A,B, comprehensive database mining identified 795 ALI therapeutic targets and 1768 bioactive component targets from PR‐HS. Intersection analysis revealed 239 overlapping targets (30.06% of total ALI‐associated targets). A PPI network was constructed (confidence score > 0.9) and visualized, revealing 258 nodes and 781 edges (Figure 1C,D). Topological parameters identified 30 core targets (e.g., STAT3, TNF, NF‐κB, TLR4) with high connectivity. Phytochemical screening classified the bioactive components into five primary classes (Figure 1E): flavonoids (44.74%), lipids (21.05%), terpenoids (21.05%), alkaloids (5.26%), and others (5.26%). The drug‐compound‐target‐disease network diagram (258 nodes, 781 edges; Figure 1F) indicated that 16 active ingredients in PR‐HS target 239 ALI‐related proteins. Flavonoids constituted the majority (9 out of 16) of these active components, with representatives such as daidzin, quercetin, and β‐Methoxydaidzein. Based on this predominance and their established relevance to liver protection, flavonoids were prioritized as key candidates for further investigation in process refinement and efficacy validation. Gene Ontology (GO) enrichment analysis identified the involvement of key biological processes (BPs), including response to molecules of bacterial origin, response to lipopolysaccharide, and regulation of inflammatory response (Figure 1G). Functional enrichment analysis via Kyoto Encyclopedia of Genes and Genomes (KEGG) uncovered significant enrichment in pathways such as Toll‐like receptor signaling, Th1 and Th2 cell differentiation, and NF‐κB signaling (Figure 1H).

### 3.2 *Lp. plantarum* and *L. paracasei* Co‐Fermentation Enhances the Bioactivity of PR‐HS

**FIGURE 2:**
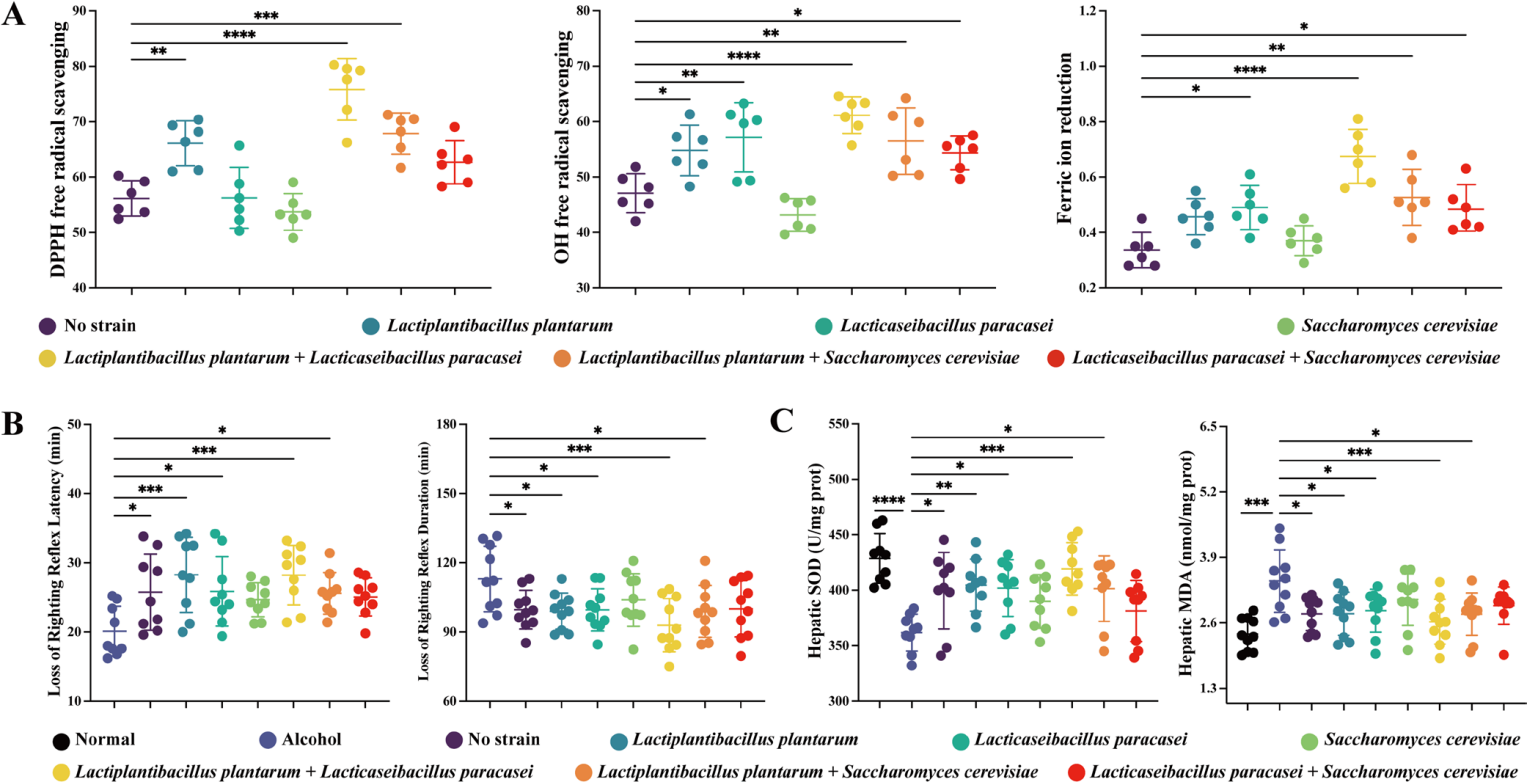
Co‐fermentation of PR‐HS with *Lp. plantarum* and *L. paracasei* demonstrated optimal antioxidant activity and alcohol‐resistance capacity. (A) Comparative analysis of DPPH free radical scavenging capacity, OH free radical scavenging capacity, and ferric ion‐reducing capacity with different strains (*n* = 6); (B) Comparative analysis of latency to loss of righting reflex, recovery duration from alcohol intoxication, (C) hepatic antioxidant: Superoxide dismutase (SOD) activity, and malondialdehyde (MDA) levels in mice (*n* = 10). Data: means ± SD. Statistical analysis: One‐way ANOVA with Dunnett's post hoc test (*p < 0.05, **p < 0.01, and ***p < 0.001 vs. Alcohol group).

Comparison of the in vitro and in vivo activities of PR‐HS treated with different strains revealed that co‐fermentation with two strains resulted in superior DPPH radical scavenging, OH radical scavenging, and ferric ion‐reducing capacities compared to monoculture fermentation (Figure 2A). Taking into account the latency to alcohol‐induced LORR (time interval between alcohol administration and LORR onset) and recovery duration from alcohol intoxication (time interval between LORR onset and righting reflex recovery) in alcohol‐induced mice (Figure 2B), coupled with the finding that the combined treatment with *Lp. plantarum* and *L. paracasei* significantly reversed hepatic SOD and MDA levels in alcoholic mice (Figure 2C; *p* < 0.001), it was determined that PR‐HS treated with the combination of *Lp. Plantarum* and *L. paracasei* exhibited the highest antioxidant activity among the dual‐strain groups.

### 3.3 Co‐Fermentation Enhances Flavonoid Enrichment and Alters the Physicochemical Profile

**FIGURE 3:**
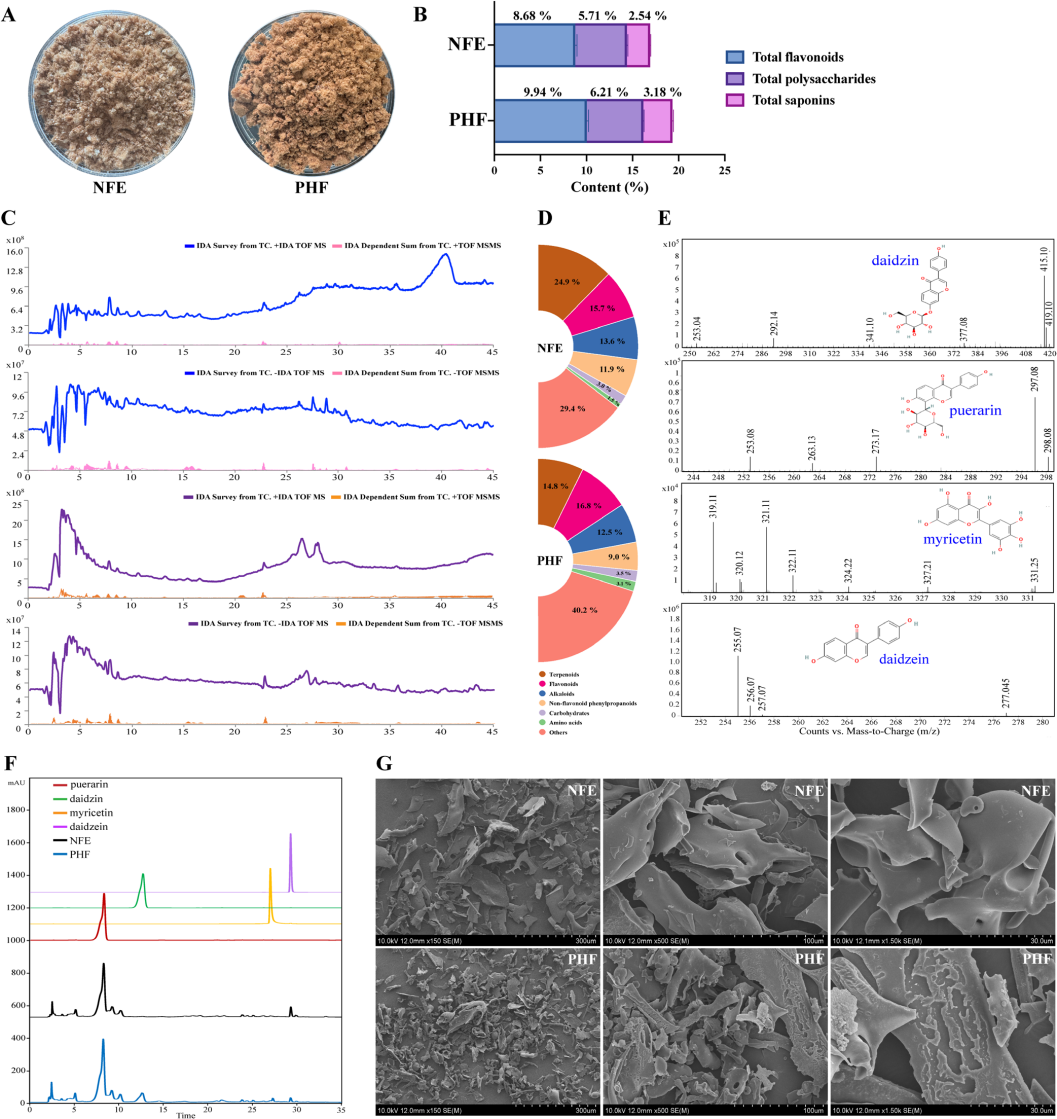
Effect of co‐fermentation on the component distribution and microstructure of PR‐HS. (A) The freeze‐dried powders of the 60% ethanol extracts from unfermented PR‐HS (NFE) and the 60% ethanol extracts from fermented PR‐HS (PHF); (B) component analysis of total flavonoids, total polysaccharides, and total saponins; (C) HPLC‐Q/TOF‐MS total ion chromatograms (TICs) in positive and negative ion mode; (D) compound classification using the Agilent TCM library of NFE and PHF; (E) mass spectrometry chromatograms of daidzin, puerarin, myricetin, and daidzein identified in PHF; (F) fermentation altered the flavonoids profile, as evidenced by HPLC, with elevated levels of daidzin, puerarin, and myricetin, and a reduction in daidzein content; (G) representative SEM images of residues from NFE and PHF groups (scale bar = 300, 100, 30 μm).

NFE and PHF powders were obtained from the mixed herbal powder, with yields of 18.14% ± 0.21% and 26.26% ± 0.29% (Figure 3A). PHF was a brown lyophilized powder containing 9.94% ± 0.17% total flavonoids, 6.21% ± 0.10% total polysaccharides, 3.18% ± 0.10% total saponins, which were 14.48% ± 0.98%, 8.88% ± 0.70%, and 25.14% ± 1.89% higher than those in NFE, respectively (Figure 3B). The determination methods are described in Methods S1–S3. HPLC‐Q‐TOF‐MS analysis was further employed to validate the key components predicted by network pharmacology. The corresponding total ion chromatograms (TICs) are presented in Figure 3C. In positive mode, both NFE and PHF had intense peak clusters at 35–45 min with distinct profiles, indicating abundant positively ionizable components (e.g., protonated alkaloids, glycosides (M + H)^+^) in both, but with differences in type/content. In negative mode, PHF had more dispersed peaks than NFE, suggesting it contains more diverse/abundant negatively ionizable components (e.g., phenolics, flavonoids as (M−H)^−^) than NFE. Through database matching, 337 and 256 metabolites were identified from NFE and PHF, respectively (Figure 3D). Classification analysis revealed that flavonoids accounted for 16.8% of the total metabolites in PHF, followed by terpenoids and alkaloids. Metabolites with an ID score of 0.95 or higher are listed in Table S2. Further analytical characterization identified four distinct flavonoid compounds in PHF (Figure 3E). *m*/*z* 415.10 and 253.04 may correspond to (M‐H)^−^ and (M‐H‐C_6_H_10_O_5_)^−^ fragments of daidzin (416.1). *m*/*z* 253.08 and 297.08 may be (M‐H‐C_6_H_10_O_5_)^−^ and (M‐H‐C_4_H_8_O_4_)^−^ fragments of puerarin (416.1). *m*/*z* 319.11 and 331.25 may represent (M + H)^+^ and (M + Na)^+^ fragments of myricetin (318.2), while *m*/*z* 255.06 and 277.05 may be (M + H)^+^ and (M + Na)^+^ fragments of daidzein (254.1). Similarly, the most abundant flavonoids in PHF analyzed by HPLC were puerarin (3.19% ± 0.02%), followed by daidzin (1.57% ± 0.01%), myricetin (1.04% ± 0.03%), and daidzein (0.43% ± 0.01%). Compared with the NFE, the contents of puerarin, myricetin, and daidzin in PHF increased by 19.70% ± 0.21%, 84.41% ± 0.34%, and 694.90% ± 15.29%, respectively, whereas the content of daidzein decreased by 48.33% ± 0.64% (Figure 3F). Morphological analysis revealed that PHF particles were finer and more than those of NFE, suggesting that fermentation degraded macromolecular substances or polymer aggregates. Furthermore, the surface of PHF showed significantly increased roughness, with substantial erosions and pits, indicating that surface etching occurred during fermentation (Figure 3G). In summary, the fermentation process alters the chemical profile of the PR‐HS extract and elevates the levels of key flavonoids, which are its major bioactive components.

### 3.4 PR‐HS Co‐Fermentation Extracts Promote Alcohol Sobriety and Hepatic Ethanol Metabolism in Mice

**FIGURE 4:**
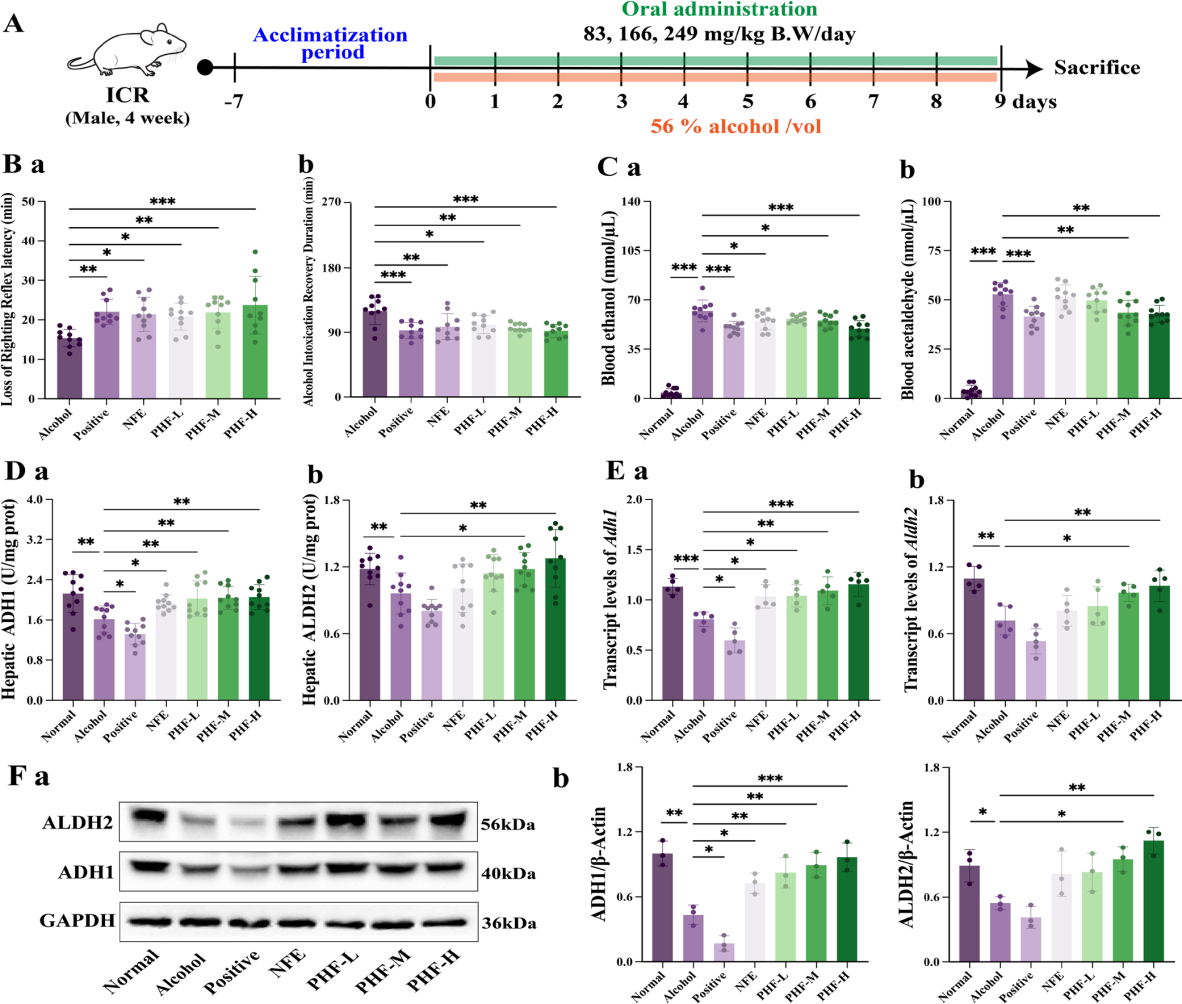
PHF promotes alcohol sobriety and hepatic ethanol metabolism in mice. (A) Schematic diagram in mice during 9‐days alcohol feeding; (B) PHF prolongs the latency to loss of righting reflex, while shortening the recovery duration from alcohol intoxication; (C) Blood ethanol and acetaldehyde, (D) Hepatic alcohol dehydrogenase 1 (ADH1; a) and acetaldehyde dehydrogenase 2 (ALDH2; b) levels were increased by PHF (*n* = 10); (E) The transcript levels of *Adh1* (a) and *Aldh2* (b) in liver were detected (*n* = 5); (F) Immunoblot (a) and quantification (b) of ADH1 and ALDH2. Gray values were normalized to GAPDH (*n* = 3). Data: means ± SD. Statistical analysis: One‐way ANOVA with Dunnett's post hoc test (*p < 0.05, **p < 0.01, and ***p < 0.001 vs. lcohol group).

The effect of PHF on anti‐alcohol sobriety was investigated by establishing an alcohol‐induced mice alcoholism model (Figure 4A). Evaluation of anti‐intoxication efficacy revealed that PHF dose‐dependently prolonged the LORR in mice (Figure 4B) while significantly shortening the recovery duration (time interval between LORR onset and righting reflex recovery). Blood ethanol and acetaldehyde concentrations in the PHF group were lower than those in the Alcohol group (Figure 4C). Expression levels and enzyme activity of key enzymes (ADH1, ALDH2) were determined to assess the effect of PHF on ethanol metabolism in mice. PHF significantly counteracted the alcohol‐induced reduction in ADH1 and ALDH2 enzyme activities in the livers of mice with ALI (*p <* 0.001) (Figure 4D). Moreover, PHF intervention increased in mRNA and protein expressions of ADH1 and ALDH2, with the high‐dose PHF (PHF‐H) showing a more pronounced effect than NFE (Figure 4E,F). These results suggest that PHF enhances the capacity of liver tissue to metabolize ethanol.

### 3.5 PR‐HS Co‐Fermentation Extracts Relieve Alcoholic Hepatoxicity in Mice

**FIGURE 5:**
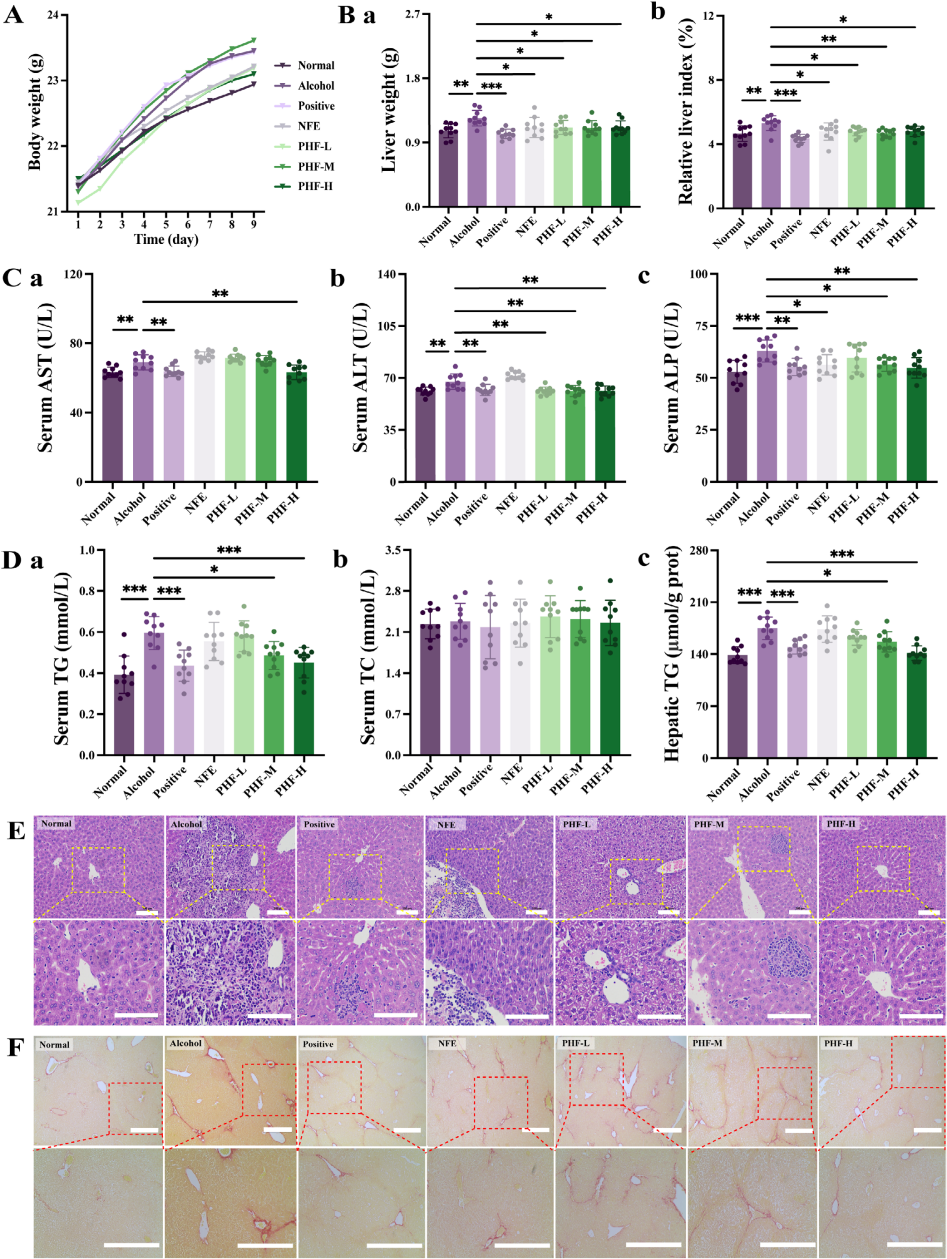
PHF relieves hepatoxicity in alcohol‐exposed mice. (A, B) Body weight trajectories, liver weight, and relative liver index are measured within 9 days; (C) PHF decreases serum AST, ALT, and ALP levels; (D) Serum TG, TC, and hepatic TC levels are measured; (E) H&E staining of liver tissue; scale bar, 100 μm. (F) Sirius red staining of liver tissue; scale bar, 400 μm. Type I collagen fibers show red, and type IV collagen fibers show pale yellow. Data: Means ± SD (*n* = 10). Statistical analysis: One‐way ANOVA with Dunnett's post hoc test (*p < 0.05, **p < 0.01, and ***p < 0.001 vs. Alcohol group).

No significant difference in body weight was observed between the PHF‐treated animals and those in the Alcohol group (Figure 5A). Compared to the Alcohol group, PHF treatment significantly decreased liver weight and relative liver index (liver weight/body weight) (Figure 5B). Moreover, the PHF‐H group exhibited substantially lower serum levels of aspartate aminotransferase (AST), alanine aminotransferase (ALT), and alkaline phosphatase (ALP) than the Alcohol group (*p* < 0.01; Figure 5C). PHF administration reduced both serum and hepatic triglycerides (TG), although no significant change was observed in serum total cholesterol (TC; Figure 5D). According to the H&E and Sirius Red staining (Figure 5E,F), the Alcohol group exhibited characteristic pathological alterations, including steatosis, hepatocellular disarray, inflammatory cell infiltration, hepatocyte necrosis, loss of hepatocytes, and the expansion of collagen fibers from perivascular areas, with abundant red‐stained type I and yellow‐stained type IV collagen fibers. In contrast, the PHF group showed a makered reduction in inflammatory cell infiltration and fibrous tissue hyperplasia, leading to effective alleviation of liver cell injury—an effect that was more pronounced than that of NFE. In summary, PHF provides significant protection against alcoholic hepatocellular injury.

### 3.6 PR‐HS Co‐Fermentation Extracts Attenuate Hepatic Inflammation by Modulating TLR4/MyD88/NF‐κB Signaling in ALI Mice

**FIGURE 6:**
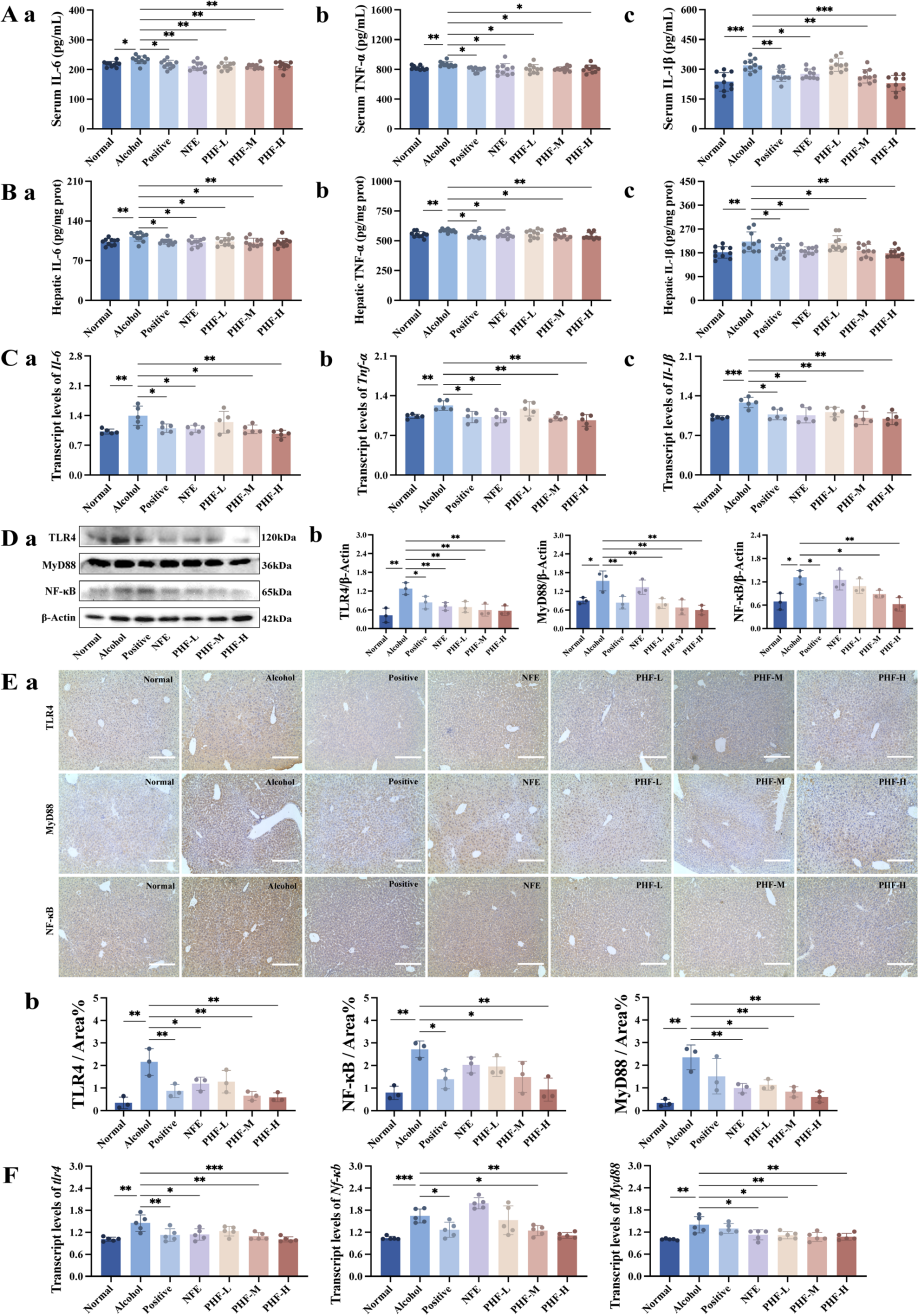
PHF suppresses proinflammatory factors and downregulates TLR4/MyD88/NF‐κB overexpression in ALI mice. (A) Serum IL‐6, TNF‐α, and IL‐1β (*n* = 10); (B) hepatic IL‐6, TNF‐α, and IL‐1β (*n* = 10); (C) the mRNA levels of *Il‐6*, *Tnf‐α*, and *Il‐1β* in livers (*n* = 5); (D) immunoblot (a) and quantification (b) of TLR4, NF‐κB, and MyD88 in livers. Gray values were normalized to β‐Actin (*n* = 3); (E) immunohistochemistry analysis (a) and semi‐quantitation (b) in livers (*n* = 3). Hematoxylin‐stained nucleus shows blue color and positive signal of DAB staining shows brown‐yellow color; scale bar, 400 μm. (F) The mRNA levels of *Tlr4*, *Nf‐κb*, an*d Myd88* in livers of mice (*n* = 5). Data: means ± SD. Statistical analysis: One‐way ANOVA with Dunnett's post hoc test (*p < 0.05, **p < 0.01, and ***p < 0.001 vs. lcohol group).

As network pharmacology and pathological analyses in mice liver have shown, ALI is often accompanied by an inflammatory response. The serum levels of IL‐6, TNF‐α, and IL‐1β in the PHF‐M and PHF‐H groups were significantly lower than those in the Alcohol group (Figure 6A). Similarly, they also reduced hepatic IL‐6, TNF‐α, and IL‐1β (Figure 6B). PHF‐M and PHF‐H downregulated the mRNA expression levels of *Il‐6*, *Tnf‐α*, and *Il‐1β* in alcohol‐exposed mice (Figure 6C). Moreover, PHF‐H exhibited a stronger suppressive effect on these inflammatory cytokines (IL‐6, TNF‐α, and IL‐1β) compared to NFE. These findings suggest that PHF ameliorates hepatic inflammation in mice with ALI.

The PPI core targets indicated the regulation of TLR4 receptor activation by PHF, with the dominant MyD88‐dependent pathway and downstream NF‐κB signaling being simultaneously validated. As expected, PHF significantly downregulated the protein expression levels of TLR4, MyD88, and NF‐κB in liver tissue (Figure 6D). Consistent results were obtained through immunohistochemical analysis of liver (Figure 6E). Furthermore, qRT‐PCR showed inhibition of *Tlr4*, *Myd88*, and *Nf‐κb* mRNA levels in PHF groups, with the highest dose reducing expression by 2‐fold compared to the Alcohol group (*p* < 0.01; Figure 6F). Notably, PHF exhibited more potent regulatory effects on TLR4 and MyD88 expression compared to NFE, while NFE did not significantly influence NF‐κB expression levels. It demonstrated that PHF downregulated the expression of TLR4, MyD88, and NF‐κB at both protein and mRNA levels, leading to suppressed pro‐inflammatory cytokine production.

### 3.7 PR‐HS Co‐Fermentation Extracts Regulate Molecular Expression of IL‐10 and SOCS1/3, Key Regulators of Macrophage Polarization

**FIGURE 7:**
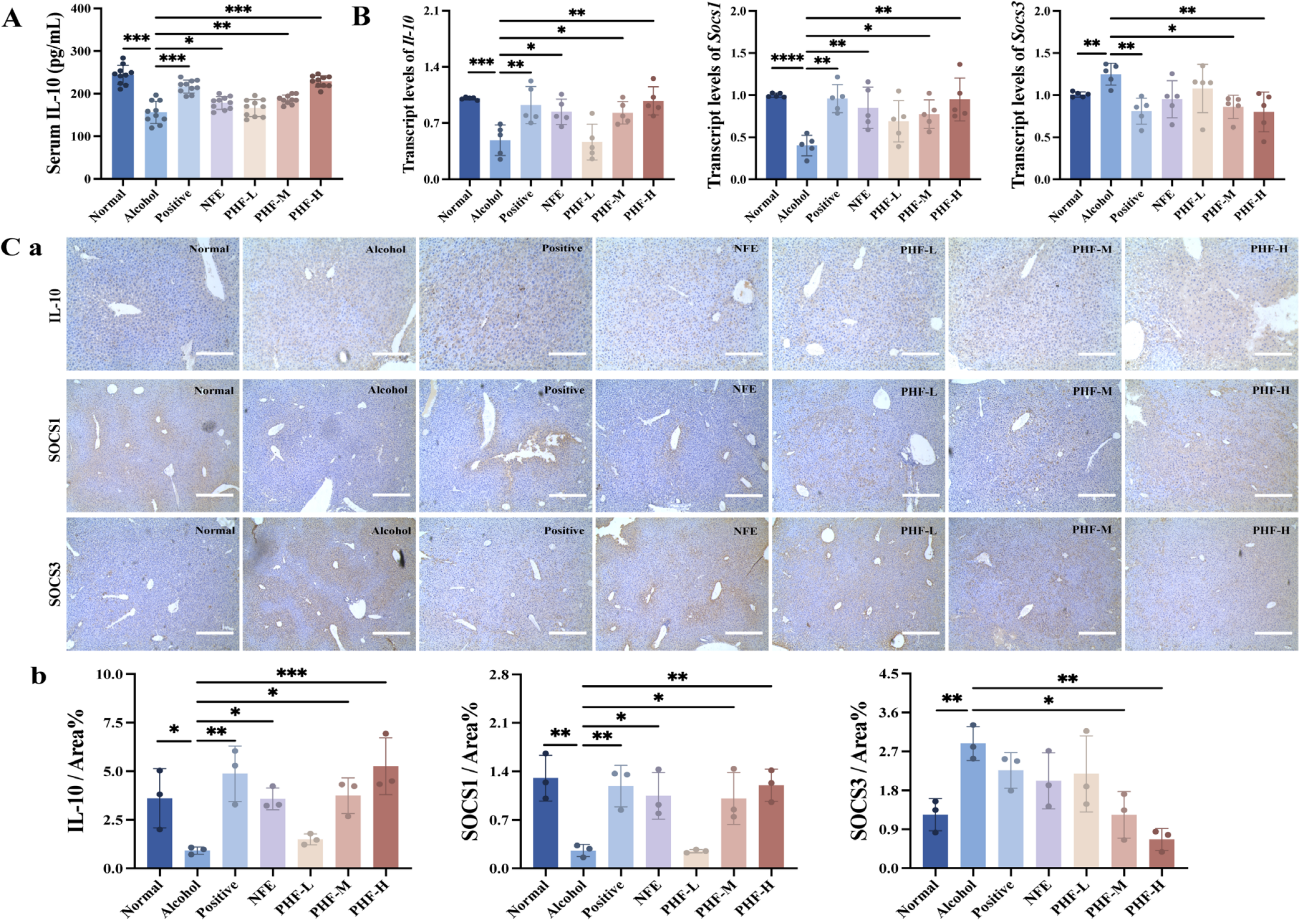
PHF upregulates the expression (both mRNA and protein) of key macrophage polarization markers IL‐10 and SOCS1/3 in the livers of ALI mice. (A) PHF increases the level of serum IL‐10 in alcohol‐exposed mice (*n* = 10); (B) The mRNA expression of *Il‐10*, *Socs1*, and *Socs3* was analyzed (*n* = 5); (C) Immunohistochemical analysis of protein expression levels of IL‐10, SOCS1, and SOCS3 in liver (*n* = 3). Hematoxylin‐stained nucleus shows blue color and positive signal of DAB staining shows brown‐yellow color; scale bar, 400 μm. Data: means ± SD. Statistical analysis: One‐way ANOVA with Dunnett's post hoc test (*p < 0.05, **p < 0.01, and ***p < 0.001 vs. Alcohol group).

Macrophage polarization controls the inflammatory balance by regulating key signaling pathways, including the JAK/STAT and TLR/NF‐κB pathways. This regulation is tightly modulated by multiple signaling molecules, such as the major regulators SOCS1/SOCS3 (key members of the SOCS family) and IL‐10 (an anti‐inflammatory cytokine). Testing this hypothesis in mice with ALI found that the serum IL‐10 levels in PHF‐H were significantly higher than that in Alcohol (*p* < 0.001; Figure 7A). Compared with the Alcohol group, PHF‐H upregulated *Il‐10* and *Socs1* mRNA by ~2‐fold and downregulated *Socs3* mRNA by ~2‐fold (Figure 7B). Further immunohistochemical analysis of liver revealed significantly upregulated protein expression levels of IL‐10 and SOCS1, while SOCS3 was markedly downregulated compared to the Alcohol group (Figure 7C). Notably, NFE showed no significant reduction in SOCS3 protein expression levels. Based on the findings of the preceding experiments, PHF may reduce hepatic inflammation by regulating macrophage polarization.

### 3.8 PR‐HS Co‐Fermentation Extracts Regulate Macrophage Polarization in RMCs Primed With LPS

**FIGURE 8:**
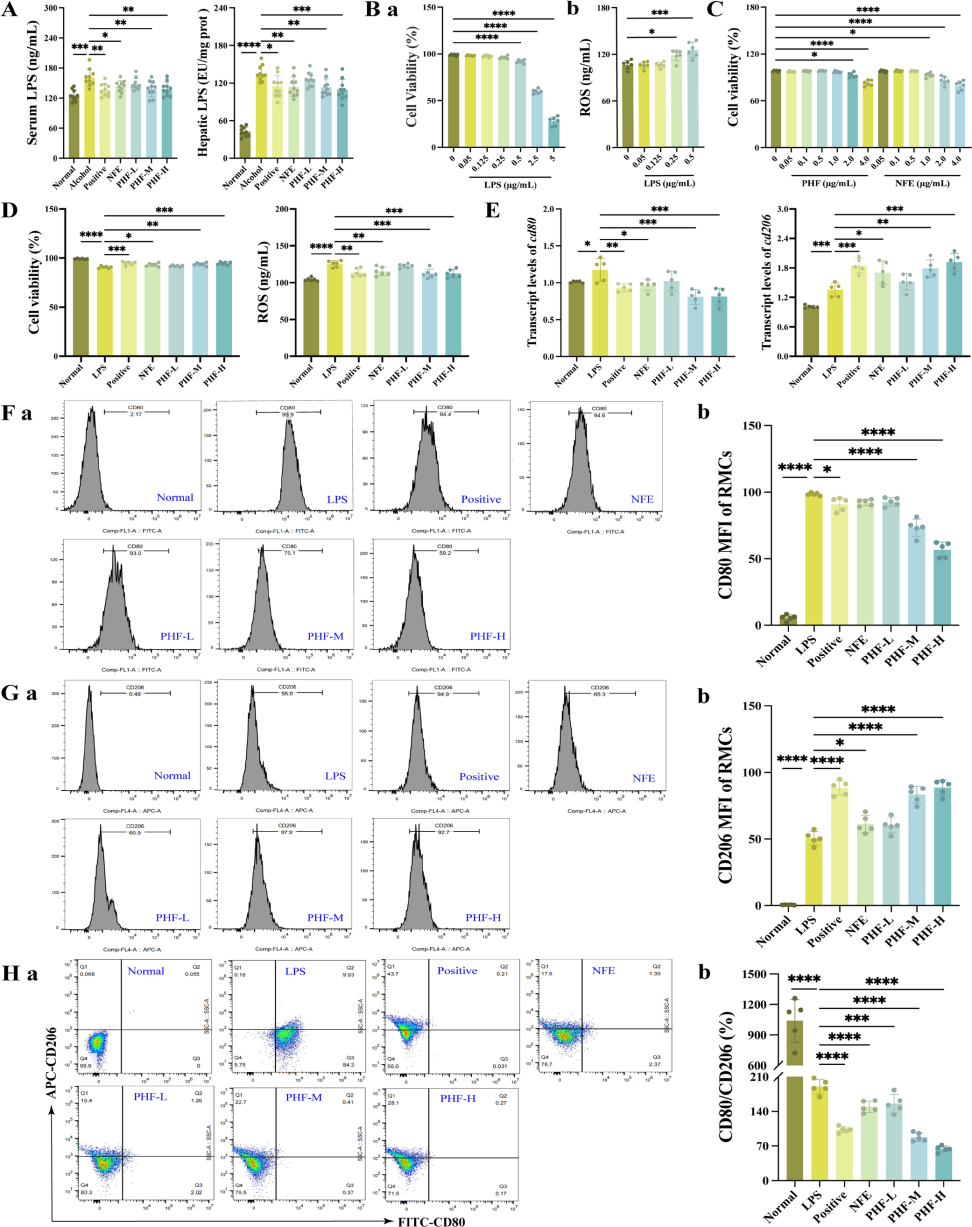
PHF regulates RAW264.7 macrophage cells (RMCs) polarization and promotes M1 phenotype conversion to M2 phenotype. (A) PHF increases the level of serum and hepatic lipopolysaccharide (LPS) in alcoholic liver injury mice (*n* = 10); (B) LPS reduced cell viability and increased reactive oxygen species (ROS) production in RMCs; (C) neither PHF (< 1.0 μg/mL) nor NFE (< 1.0 μg/mL) exhibited cytotoxicity; (D) PHF enhanced the viability of M1‐polarized macrophages and reduced ROS levels (*n* = 6); (E) PHF decreased the transcriptional levels of CD80 (M1 macrophages) while increasing that of CD206 (M2 macrophages); (F, G) PHF attenuated activation of CD80 while promoting activation of CD206; (H) PHF demonstrated super efficacy in reducing the M1/M2 ratio (*n* = 5). Data: Means ± SD. Statistical analysis: One‐way ANOVA with Dunnett's post hoc test (*p < 0.05, **p < 0.01, ***p < 0.001, and ****p < 0.0001 vs. LPS group).

To investigate the occurrence of macrophage polarization, which is closely associated with hepatic inflammation, and to characterize macrophage subtypes, an M1‐polarized macrophage model was established. Serum and hepatic (Figure 8A) LPS levels were significantly elevated in ALI mice while PHF significantly reduced serum and hepatic LPS levels, suggesting a positive correlation between LPS levels and disease severity. Consequently, LPS was employed to induce macrophage polarization and establish an M1 phenotype model. MTT assay revealed that cell viability began to drop below 90% when LPS exceeded 0.5 μg/mL (Figure 8Ba). RMCs treated with LPS at 0.25 and 0.5 μg/mL exhibited significantly elevated reactive oxygen species (ROS) levels compared to the Normal group, indicating successful establishment of the M1‐polarized macrophage model (Figure 8Bb). Neither PHF (< 1.0 μg/mL) nor NFE (< 1.0 μg/mL) exhibited cytotoxicity (Figure 8C). Moreover, both treatments significantly enhanced the viability of LPS (0.5 μg/mL)‐induced M1‐polarized macrophages and reduced ROS levels (*p* < 0.001; Figure 8D). Notably, PHF‐M demonstrated a stronger pro‐proliferative effect than NFE at the same concentration. Compared to LPS group, PHF attenuated the transcriptional levels of pro‐inflammatory M1 macrophage marker (CD80) while promoting that of anti‐inflammatory M2 macrophage marker (CD206) (Figure 8E). Flow cytometry showed that the mean fluorescence intensity (MFI) of M1 phenotype was significantly decreased (Figure 8F), while the MFI of M2 phenotype was markedly increased after treatment with PHF and NFE (Figure 8G). Similarly, PHF demonstrated superior efficacy in reducing the M1/M2 ratio compared to NFE at equivalent concentrations (Figure 8H). Briefly, PHF affects macrophage polarization and promotes M1 phenotype conversion to M2 phenotype.

### 3.9 PR‐HS Co‐Fermentation Extracts Mediate Macrophage Phenotype via Modulation of Polarization‐Related Gene and Protein Biomarkers in LPS‐Induced M1‐Polarized RMCs

**FIGURE 9:**
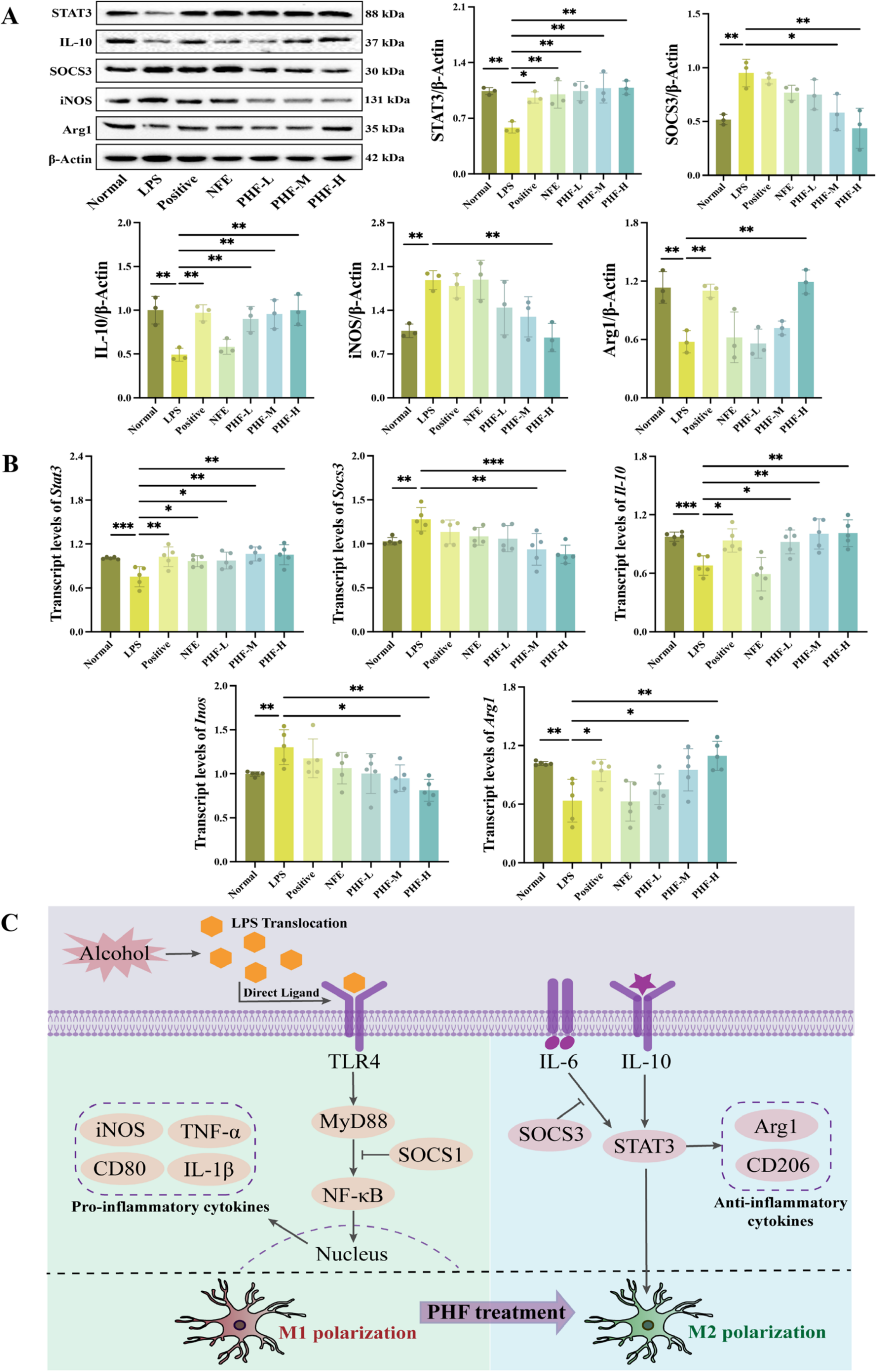
PHF attenuates LPS‐induced M1‐polarized RMCs activation toward M1 phenotype and tend to M2 phenotype. (A) Immunoblot and quantification of STAT3, SOCS3, IL‐10, iNOS, and Arg1. Gray values were normalized to β‐Actin (*n* = 3); (B) mRNA levels of *Stat3*, *Socs3*, *Il‐10*, *inos*, and *Arg1* (*n* = 5). Data: Means ± SD. Statistical analysis: One‐way ANOVA with Dunnett's post hoc test (*p < 0.05, **p < 0.01, and ***p < 0.001 vs. aLPS group). (C) Proposed mechanism: Alcohol activates TLR4, triggering the MyD88‐dependent pathway to promote nuclear translocation of NF‐κB, thereby stimulating the release of pro‐inflammatory mediators and enhancing M1 macrophage polarization. In contrast, PHF suppresses the TLR4/MyD88/NF‐κB signaling pathway while upregulating the IL‐10/STAT3 axis to induce M2 macrophage differentiation, promoting the secretion of anti‐inflammatory factors.

The effect of PHF on the regulation of macrophage polarization signaling pathways in M1‐polarized RMCs was further investigated, focusing on the downstream mediator of TLR4 pathway. Treatment with the PHF‐H exhibited significantly downregulated the protein expression of STAT3 and SOCS3, while markedly increasing IL‐10 levels (Figure 9A). Concurrently, PHF‐H downregulated the expression of iNOS (an M1 phenotype biomarker) and upregulated that of Arg1 (an M2 phenotype biomarker) in RMCs. Notably, NFE exhibited regulatory effects on STAT3 and IL‐10 that were comparable to those of PHF, but its effects on SOCS3, iNOS, and Arg1 were less pronounced. Correspondingly, the mRNA expression patterns of macrophage polarization markers (*Stat3*, *Socs3*, *Il‐10*, *Inos*, and *Arg1*) were consistent with their respective protein levels (Figure 9B). These results demonstrate that the ameliorative effects of PHF on LPS‐induced M1‐polarized RMCs are associated with attenuated M1 phenotype activation and a shift toward M2 phenotype.

## 4 Discussion

Numerous Chinese medical texts show that PR‐HS are effective alcohol detoxification medications, leading to their widespread use in the health food industry. Network pharmacology analysis suggests that flavonoids in PR‐HS make a significant contribution to anti‐ ALI effects. This study extracted flavonoids from botanical drugs using ethanol, following the polar phase dissolution principle (Liu, Yuan, et al. 2021; Vo Van et al. 2022). Pre‐screening experiments indicated that co‐fermentation with *Lp*. *plantarum* and *L. paracasei* was associated with stronger alcohol‐detoxifying and antioxidative effects in mice than fermentation with either strain alone or with *S. cerevisiae* . In parallel, post‐fermentation PHF displayed altered chemical characteristics, including higher daidzin and lower daidzein levels, and showed better alcohol‐counteracting and hepatoprotective effects than unfermented NFE. These observations support the hypothesis that fermentation‐driven compositional changes underlie the enhanced bioactivity. A plausible mechanism is microbially mediated biotransformation: certain lactic acid bacteria, including Lp. plantarum and *L. paracasei* , have been reported to display glycosyltransferase‐related activity that could convert daidzein to daidzin in the presence of suitable glycosyl donors (London et al. 2014; Nguyen et al. 2021; Razola‐Diaz et al. 2024; Soumya et al. 2023; Xiao et al. 2021; Zhang et al. 2025). Such bioconversion may offer a dual advantage: (1) it preserves the glycoside form, which exhibits enhanced aqueous solubility and may possess intrinsic bioactivity; and (2) it modulates the ratio of glycoside to aglycone, potentially refining the bioactivity spectrum to favor alcohol detoxification and antioxidant effects over estrogenic activity (Chen, Chang, et al. 2022; Hwang et al. 2006; Qiang et al. 2022; Sajid et al. 2021; Sittipo et al. 2019). However, current evidence is correlative. Definitive causal links require targeted follow‐up, for example in vitro biotransformation assays with isolated strains and substrates, enzyme identification (glycosyltransferase gene/protein characterization), isotopic tracing, and in vivo pharmacology using purified daidzin and daidzein to quantify their individual contributions. In addition to compositional changes, fermentation may improve formulation properties relevant to bioavailability and safety by reducing particle size, producing a more homogeneous dispersion, degrading nontarget matrix components, and exposing polar functional groups that enhance solubility and molecular interactions (Chi et al. 2025; Zhao et al. 2025). In our process optimization experiments, a neutral pH (7.0) and 2% inoculum increased flavonoid yields, highlighting the importance of pH and strain density for lactic acid bacterial growth and metabolism. Collectively, these results indicate that controlled fermentation can increase flavonoid content and potentially improve bioavailability of PR and HS extracts, but mechanistic validation and comprehensive pharmacokinetic and safety studies are needed before clinical translation (Ruiz de la Bastida et al. 2021).

Alcohol‐induced liver injury is commonly characterized by hepatic lipid accumulation, an increased liver index, and elevated serum transaminases. When hepatocellular damage occurs, altered membrane permeability promotes the release of ALT and AST into the circulation, making these enzymes useful indicators of liver injury (Cao et al. 2023; Dong et al. 2020; Teixeira et al. 2023). In the present study, PHF showed greater efficacy than NFE in reducing the relative liver index, lowering serum ALT and AST levels, and alleviating histopathological damage, indicating a stronger hepatoprotective effect. A plausible explanation for this difference is the altered isoflavone profile after fermentation, particularly the increase in daidzin and decrease in daidzein. Importantly, the superior effect of PHF does not necessarily mean that daidzin is intrinsically more active than daidzein. Rather, the two compounds may differ in absorption and metabolic behavior in vivo. Due to its greater lipophilicity, daidzein can be absorbed more rapidly in the small intestine, potentially leading to a relatively rapid increase and decline in systemic exposure. By contrast, daidzin is more hydrophilic and is less readily absorbed in its intact form. It may reach the colon and then be hydrolyzed by microbial β‐glucosidases to release daidzein, thereby producing a delayed or more prolonged exposure profile (Moreira et al. 2023; Sittipo et al. 2019; Yoshikata et al. 2018; Zhang et al. 2011). This difference may help explain why PHF provided better protection against persistent alcohol‐induced injury (Alqudah and Claesen 2024; Baciu et al. 2023; Qiang et al. 2022). Histopathological observations further supported the biochemical findings. Excessive alcohol exposure is known to induce hepatocyte swelling, vacuolar degeneration, lipid accumulation, inflammatory injury, and, in severe cases, fibrotic changes. In our study, PHF treatment mitigated these pathological alterations, as reflected by improved hepatic architecture and reduced hepatocyte necrosis. Nevertheless, the proposed mechanism linking the daidzin/daidzein shift to enhanced hepatoprotection remains inferential. Further pharmacokinetic, gut microbiota–dependent, and compound‐specific efficacy studies are needed to verify whether the altered isoflavone composition is causally responsible for the superior activity of PHF.

ADH is predominantly expressed by hepatocytes, and it is responsible for the conversion of the majority of ethanol to acetaldehyde. Activated ALDH enters mitochondria, where it converts acetaldehyde to acetic acid (Yan et al. 2023). Consequently, the accumulation of deleterious metabolites such as acetaldehyde and alcohol in liver tissues may lead to hepatotoxicity eventually. PHF treatment increased the number of ethanol metabolism‐specific enzymes while decreasing the levels of ethanol and acetaldehyde metabolites. Furthermore, daidzin—a traditional anti‐alcoholism component—has been shown to modulate alcohol metabolism by competitively inhibiting ALDH2. Pretreatment with daidzin reduced acetaldehyde accumulation and attenuated signs of intoxication in mice (Lowe et al. 2008; Wang et al. 2022). This evidence aligns with and supports the proposed mechanism in our study, wherein fermentation enhances anti‐intoxication efficacy. Notably, distinct from Puerarin Schisandra tablets, PHF exhibits a characteristic feature of directly activating the key alcohol‐metabolizing enzymes, ADH and ALDH2. This mechanism accelerates ethanol clearance at its source, thereby demonstrating unique advantages in both alcohol‐sobering and hepatoprotective activities.

Network pharmacology analysis suggests that PR‐HS may alleviate ALI via the TLR signaling pathway and NF‐κB signaling pathways, a hypothesis subsequently verified in our alcohol‐exposed mouse model. Ethanol exposure can compromise the intestinal barrier, leading to the translocation of gut‐derived microbial components, such as LPS and β‐glucan, to the liver. This process fosters inflammation and fibrosis in ALI (Duan et al. 2019; Irizarry‐Caro et al. 2020). In the liver, these pathogen‐associated molecular patterns (PAMPs) bind to pattern recognition receptors, notably the TLR4 complex on macrophages, which serve as a first line of defense. This binding triggers the release of pro‐inflammatory mediators, which subsequently activate other innate and adaptive immune cells. Specifically, the LPS‐TLR4 interaction initiates MyD88‐dependent signaling, leading to NF‐κB pathway activation and the release of pro‐inflammatory cytokines, thereby driving ALI progression (Wen et al. 2022). Activation of the TLR4/MyD88/NF‐κB axis elevates the production of key inflammatory cytokines, including TNF‐α, IL‐6, and IL‐1β, culminating in a pronounced immune‐inflammatory response (Hritz et al. 2008). Consistent with this mechanism, PHF treatment suppresses the alcohol‐induced activation of the TLR4/MyD88/NF‐κB signaling pathway in mice, resulting in reduced hepatic pro‐inflammatory cytokine levels and the amelioration of liver injury. The proposed mechanism of action is summarized in Figure 9C.

IL‐10 is a multifunctional immunomodulatory cytokine produced by TLR‐activated macrophages. It aids antigen presentation, reduces inflammation, protects the host, and helps maintain immune homeostasis. As an essential inflammatory modulator, IL‐10 exerts innate anti‐inflammatory effects partly by inducing Arg1 expression in macrophages and regulating the shift from M1 to M2 polarization, thereby suppressing excessive inflammation and promoting tissue repair (Hu et al. 2022; Saraiva and O'Garra 2010). On the other hand, TLR activation in macrophages also triggers the induction of SOCS proteins. Studies indicate that IL‐10 promotes the anti‐inflammatory M2 phenotype by increasing the SOCS1/SOCS3 ratio, which can serve as a potential M2 marker. Conversely, a higher SOCS3/SOCS1 ratio is associated with pro‐inflammatory M1 polarization. As negative feedback regulators, SOCS proteins modulate STAT‐mediated signaling, with the STAT‐SOCS axis playing a critical role in macrophage polarization (Liu, Chen, et al. 2021; Morris et al. 2018; Witalisz‐Siepracka et al. 2022). In this study, PHF promoted macrophage polarization toward an immunoregulatory phenotype by upregulating IL‐10 and modulating SOCS1/3 expression, thereby influencing NF‐κB signaling. The transition from an M1 to an M2 phenotype is likely promoted by PHF‐stimulated endogenous IL‐10, which reduces SOCS3 expression and increases the SOCS1/SOCS3 ratio.

Macrophages are crucial for regulating and resolving inflammation, as they utilize TLR members to recognize microbial structures that form pathogen‐associated molecular patterns (PAMPs) (Ariel et al. 2012; Irizarry‐Caro et al. 2020). Notably, the network pharmacology analysis highlighted the vital roles of immune response and macrophage polarization in the ALI‐alleviating effect of PR‐HS, findings that were subsequently verified using an LPS‐induced macrophage model in this study. In response to various stimuli, macrophages undergo polarization into distinct functional subtypes. Exposure to stimuli such as LPS and TNF‐α drives macrophages toward a pro‐inflammatory M1 phenotype. ROS, as one of the earliest products of macrophage activation, serve as a direct indicator of the cellular inflammatory state. In the present study, the LPS concentration for modeling was optimized based on ROS levels to ensure that macrophages maintained an appropriate and consistent state of inflammatory activation. In the context of ALI, LPS induces the production of pro‐inflammatory substances and stimulates the immune system (Chen et al. 2023; Hu et al. 2021). Flow cytometry analysis confirmed that LPS promoted M1 polarization, whereas PHF treatment shifted macrophages toward the M2 phenotype. Specifically, PHF downregulated the M1 marker iNOS and upregulated the M2 marker Arg1, confirming its ability to modulate macrophage polarization. The cytokine IL‐10 plays a key regulatory role by selectively promoting the apoptosis of M1 macrophages, partly through activating M2 macrophages to secrete Arg1, which can counter iNOS overexpression; conversely, anti‐IL‐10 antibodies reduce this pro‐apoptotic effect (Park et al. 2017; Wan et al. 2014). Thus, PHF appears to exert its reparative and hepatoprotective effects, at least in part, by redirecting macrophage polarization. Strategies that promote M2 polarization or inhibit M1 activation represent promising therapeutic approaches for preventing and treating ALI (Duncan et al. 2020; Gordon and Taylor 2005; Seitz et al. 2018).

While these findings highlight the potential of PHF as a natural therapeutic agent against ALI, several critical limitations warrant further investigation. First, the component‐function relationship of post‐fermentation PHF requires systematic characterization, particularly to identify the key bioactive constituents responsible for its superior efficacy. Second, the direct molecular targets mediating PHF's in vivo effects remain elusive. Glutathione S‐Transferase pull‐down coupled with MS could help identify covalent interaction partners, which should then be validated through mechanistic studies including gene knockout and overexpression experiments. Finally, experimental validation is needed to determine whether PHF's modulation of LPS‐driven ALI depends on its effects on intestinal barrier integrity, gut permeability, and gut microbiota composition. Addressing these questions will significantly advance the translational development of PHF as a therapeutic intervention for alcohol‐related liver damage.

## 5 Conclusion

In this study, co‐fermentation with *Lp. Plantarum* and *L. paracasei* increased the yield of *Puerariae* Radix‐*Hovenia* Seed (PR‐HS), enhanced the contents of major bioactive components—including puerarin (20%), daidzin (695%), and myricetin (84%)—and reduced macromolecular impurities while modifying the particle morphology. PHF demonstrated both in vivo and in vitro antioxidant activity and alleviated liver injury, lipid accumulation, alcohol metabolic disorders, and inflammatory cytokine secretion in ALI mice. Mechanistically, PHF modulated the TLR4/MyD88/NF‐κB signaling pathway and promoted a shift from M1 to M2 macrophage polarization, which likely contributes to the attenuation of ALI‐associated liver inflammation. These results underscore the potential of microbial co‐fermentation technology in reducing toxicity and enhancing the efficacy of functional food, thereby supporting PHF as a novel candidate for the prevention and treatment of alcoholic liver disease.

## Author Contributions

**Shen Yao:** visualization, supervision, investigation. **Daqing Zhao:** funding acquisition, resources. **Yuan Cui:** validation, methodology, data curation. **Siming Wang:** writing – review and editing, resources, methodology, formal analysis. **Shiting Yu:** writing – review and editing, resources, supervision, formal analysis. **Meichen Liu:** supervision, formal analysis. **Yunpeng Sun:** writing – original draft, project administration, investigation, data curation, conceptualization. **Yiyu Ni:** validation, software, formal analysis.

## Funding

This work was funded by National Key Research and Development Program (NKRDP) of China (No. 2021YFD1600903‐02).

## Conflicts of Interest

The authors declare no conflicts of interest.

## Supporting information


**Table S1:** Primer sequences of qRT‐PCR.
**Table S2:** Identification of the major PHF constituents.
**Methods S1:** Total flavonoid quantification.
**Methods S2:** Total polysaccharide quantification.
**Methods S3:** Total saponin quantification.

## Data Availability

The data that support the findings of this study are available from the corresponding author upon reasonable request.
