# Supplementary material for: Enhanced Bioactivity of Puerariae Radix‐ Hovenia Seed Extracts Through Lactiplantibacillus plantarum and Lacticaseibacillus paracasei Co‐Fermentation: Impact on Alcoholic Liver Injury and Macrophage Polarization
Source: Food Sci Nutr. 2026 Apr 25;14(5):e71829. doi: 10.1002/fsn3.71829 (PMC13109719; doi:10.1002/fsn3.71829)
Supplement: Supplementary file 1 — Table S1: Primer sequences of qRT‐PCR. Table S2: Identification of the major PHF constituents. Methods S1: Total flavonoid quantification. Methods S2: Total polysaccharide quantification. Methods S3: Total saponin quantification. [file FSN3-14-e71829-s001.docx]

**Supplementary Information**

Enhanced bioactivity of *Puerariae Radix*-*Hovenia Seed* extracts through *Lactiplantibacillus plantarum* and *Lacticaseibacillus paracasei* co-fermentation: Impact on alcoholic liver injury and macrophage polarization

**Supplementary data**

Methods S1. Total flavonoid Quantification

The aluminum trichloride colorimetric method was used to measure total flavonoid content. For this, 400 μL of 5 % sodium nitrite was combined with 500 μL of the sample. Then, 400 μL of 10 % aluminum nitrate and 4000 μL of 4 % caustic soda were added. The absorbance was measured at 510 nm using a microplate reader (Infinite 200 Pro, TECAN, Switzerland) after 5 minutes at ambient temperature (Infinite 200 Pro, TECAN, Switzerland). The total flavonoid quantification was conducted using a total flavonoid curve prepared with rutin (0–0.6 mg/mL, *R*^2^ = 0.9973).

Methods S2. Total polysaccharide Quantification

The total polysaccharide content was determined using the phenol-sulfuric acid method. An aliquot of the sample solution (1 mL) containing approximately 10-80 μg of carbohydrates was pipetted into a glass test tube. Then, 1 mL of a 5% (w/v) aqueous phenol solution was added, followed by the rapid addition of 5 mL of concentrated sulfuric acid. The mixture was vortexed immediately and allowed to stand for 2 min. After that, it was placed in a water bath at 80 °C for 20 min to develop the color. The absorbance of the resulting yellow-gold solution was measured at 485 nm against a blank (prepared with distilled water instead of the sample) using a spectrophotometer (Infinite 200 Pro, TECAN, Switzerland). All determinations were performed in triplicate. A standard curve was established simultaneously using glucose (Solarbio, China) over a concentration range of 0 to 100 μg/mL.

Methods S3. Total saponin Quantification

The total saponin content was determined using the vanillin-acetic acid assay. A standard stock solution was prepared by dissolving 5.0 mg of Ginsenoside Re (Sigma-Aldrich, USA) in 80% methanol and made up to a final volume of 5 mL. A series of working standard solutions were prepared by appropriate dilution. The sample extract (0.2 mL), standard solution, or blank (80% methanol) was pipetted into a glass test tube with a stopper. Then, 0.2 mL of 5% (w/v) vanillin-acetic acid solution was added, followed by the addition of 0.8 mL of perchloric acid (70%). The mixture was vortexed thoroughly and heated in a 60°C water bath for 20 min. After heating, the tubes were immediately cooled in an ice-water bath to stop the reaction. 4 mL of glacial acetic acid was added to the cooled mixture and vortexed again. The absorbance of the resulting solution was measured at 540 nm against a blank reagent using a UV-Vis spectrophotometer (Infinite 200 Pro, TECAN, Switzerland). A standard calibration curve was plotted with Ginsenoside Re concentration versus absorbance. The TSC in the samples was expressed as milligrams of Ginsenoside Re equivalents per gram of dry weight (mg RE/g dw).

Table S1. Primer sequences of qRT-PCR

| Genes | Forward (5′ to 3′) | Reverse (3′ to 5′) |
| --- | --- | --- |
| *β-actin* | GGCTGTATTCCCCTCCATCG | CCAGTTGGTAACAATGCCATGT |
| *Adh1* | **GCTGGGCTACACTGAAGCAC** | **CAGGTAGCCGAAGATGACGA** |
| *Aldh2* | GCTGGGCTACACTGAAGGAC | TCCAGGGTCTTAGCACCTTC |
| *Il-6* | GAGGATACCACTCCCAACAGACC | AAGTGCATCATCGTTGTTCATACA |
| *Tnf-α* | **CCCTCACACTCAGATCATCTTCT** | **GCTACGACGTGGGCTACAG** |
| *Il-1β* | **GCAACTGTTCCTGAACTCAACT** | **ATCTTTTGGGGTCCGTCAACT** |
| *Tlr4* | TGGATACGTTTCCTTATAAG | GAAATGGAGGCACCCCTTC |
| *Nf-κb* | **AGATCATCCGAGATGTCTGGAA** | **CGGCTTGTCACTCGGAATAC** |
| *Myd88* | **CTGCCTGGCTGGTTTACATC** | **GCCAGGATGCTGTCGATAAA** |
| *Il-10* | GCTCTTACTGACTGGCATGAG | CGCAGCTCTAGGAGCATGTG |
| *Socs1* | **CTGCGGCTTCTATTGGGGAC** | **AAAAGGCAGTCGAAGGTCTCG** |
| *Socs3* | **GCCGCCACTTACTTCCTGAC** | **GCGCAGAATCGATGCCCTTA** |
| *Cd80* | **GACCTTTGCAGCAAGAGCAAG** | **GAAAGTCAGAGCCTCCGCTTC** |
| *Cd206* | **CTCTGTTCAGCTATTGGACGC** | **CGGAATTTCTGGGATTCAGCG** |
| *Stat3* | CAGCAGCTTGACACACGGTA | AAACACCAAAGTGGCATGTGA |
| *Inos* | **CAGCTGGGCTGTACAAACCTT** | **CATTGGAAGTGAAGCGTTTCG** |
| *Arg1* | **CTCCAAGCCAAAGTCCTTAGAG** | **AGGAGCTGTCATTAGGGACATC** |

Table S2. Identification of the major PHF constituents

| RT | Analyte Name | Molecular Formula | Adduct | m/z | Measured Mass | Diff (Tgt, ppm) | Score (Tgt) |
| --- | --- | --- | --- | --- | --- | --- | --- |
| 2.337 | Puerarin | C21H20O9 | (M-H)⁻ | 415.10371 | 416.1108 | 0.27 | 98.67 |
| 2.407 | Methyl gallate | C8H8O5 | (M-H)⁻ | 183.02999 | 184.0371 | -0.38 | 95.45 |
| 3.125 | Daidzin | C21H20O9 | (M+H)⁺ | 417.11835 | 416.1111 | 0.77 | 98.44 |
| 3.194 | Trifolirhizin | C22H22O10 | (M+H)⁺ | 447.12877 | 446.1216 | 0.6 | 99.14 |
| 3.264 | Buddleoside | C28H32O14 | (M+H)⁺ | 593.18666 | 592.1795 | 0.46 | 97 |
| 3.334 | 6'''-Feruloylspinosin | C38H40O18 | (M+H)⁺ | 785.22912 | 784.2218 | 0.45 | 98.19 |
| 3.447 | Bellidifolin | C14H10O6 | (M+HCOO)⁻ | 319.04618 | 274.0479 | 0.75 | 97.71 |
| 3.794 | Soyasaponin Ba | C48H78O19 | (M-H)⁻ | 957.50644 | 958.5137 | -0.02 | 95.38 |
| 3.822 | Formononetin glucoside | C22H22O9 | (M+H)⁺ | 431.13378 | 430.1265 | 0.37 | 99.45 |
| 4.447 | Bavachin | C20H20O4 | (M+NH₄)⁺ | 342.16995 | 324.136 | -0.43 | 99.63 |
| 4.517 | Ethyl cinnamate | C11H12O2 | (M+NH₄)⁺ | 194.11765 | 176.0839 | 0.75 | 98.49 |
| 4.517 | Tetrahydrojateorrhizine | C20H23NO4 | (M+H)⁺ | 342.16995 | 341.1627 | 0.05 | 99.63 |
| 5.458 | Citrinin | C13H14O5 | (M+CH₃COO)⁻ | 309.09804 | 250.0842 | 0.16 | 99.79 |
| 5.853 | Myricetin | C15H10O8 | (M+H)⁺ | 319.04501 | 318.0377 | 0.43 | 98.21 |
| 6.686 | Indirubin | C16H10N2O2 | (M+H)⁺ | 263.08153 | 262.0743 | 0.24 | 99.76 |
| 7.379 | 2''-O-Rhamnosylvitexin | C27H30O14 | (M+H)⁺ | 579.17136 | 578.1636 | 0.15 | 99.11 |
| 7.934 | Olaquindox | C12H13N3O4 | (M+Na)⁺ | 286.07937 | 263.0904 | -0.85 | 98.12 |
| 8.212 | 4-Hydroxycinnamamide | C9H9NO2 | (M+H)⁺ | 164.07104 | 163.0637 | 2.49 | 98.43 |
| 9.688 | Bufalin | C24H34O4 | (M+HCOO)⁻ | 431.24405 | 386.246 | 0.84 | 98.09 |
| 9.945 | (+)-Gallocatechin | C15H14O7 | (M+Na)⁺ | 329.0634 | 306.0741 | 0.53 | 95.81 |
| 10.361 | Atractylodin | C13H10O | (M+NH₄)⁺ | 200.10713 | 182.0733 | 0.97 | 98.5 |
| 10.569 | Forskolin | C22H34O7 | (M+H)⁺ | 411.23791 | 410.2304 | -0.06 | 97.75 |
| 10.847 | Acetyl-trans-resveratrol | C20H18O6 | (M+H)⁺ | 355.11787 | 354.1104 | 0.23 | 97.33 |
| 10.916 | Artemisinin | C15H22O5 | (M+NH₄)⁺ | 300.18047 | 282.1467 | -0.06 | 99.28 |
| 11.123 | Groenlandicine | C19H16NO4 | (M+Na)⁺ | 345.09722 | 322.1081 | 0.36 | 99.57 |
| 11.886 | Pectolinarigenin | C17H14O6 | (M+H)⁺ | 315.08627 | 314.0791 | 0.15 | 99.8 |
| 14.658 | Acetylshikonin | C18H18O6 | (M+H)⁺ | 331.11788 | 330.1105 | 0.45 | 95.24 |
| 14.658 | Baohuoside II | C26H28O10 | (M+NH₄)⁺ | 518.20232 | 500.1685 | 0.57 | 98.86 |
| 14.797 | Androstenedione | C19H26O2 | (M+NH₄)⁺ | 304.2272 | 286.1933 | 0.08 | 96.4 |
| 16.045 | 3-n-Butylphathlide | C12H14O2 | (M+NH₄)⁺ | 208.1332 | 190.0994 | -0.06 | 99.72 |
| 16.183 | Moslosooflavone | C17H14O5 | (M+H)⁺ | 299.09128 | 298.084 | -0.3 | 99.87 |
| 18.195 | α- Asarone | C12H16O3 | (M+NH₄)⁺ | 226.14384 | 208.11 | 0.4 | 99.79 |
| 19.235 | Dihydrotanshinone I | C18H14O3 | (M+NH₄)⁺ | 296.12814 | 278.0943 | 0 | 97.35 |
| 19.305 | Fraxinellone | C14H16O3 | (M+NH₄)⁺ | 250.14379 | 232.1101 | 0.48 | 97.87 |
| 19.859 | Protopine | C20H19NO5 | (M+H)⁺ | 354.13334 | 353.1263 | -0.09 | 98.12 |
| 21.177 | Nuciferine | C19H21NO2 | (M+H)⁺ | 296.16473 | 295.1574 | 0.66 | 97.96 |
| 22.148 | Dihydrocapsaicin | C18H29NO3 | (M+H)⁺ | 308.22201 | 307.2148 | 0.11 | 99.7 |
| 22.564 | Kirenol | C20H34O4 | (M+NH₄)⁺ | 356.27943 | 338.2456 | -0.48 | 96.12 |
| 22.772 | Tectochrysin | C16H12O4 | (M+H)⁺ | 269.08084 | 268.0736 | 0.14 | 99.86 |
| 24.022 | Andrographolide | C20H30O5 | (M+H)⁺ | 351.21645 | 350.2092 | -0.44 | 98.51 |
| 24.091 | Ginkgetin | C32H22O10 | (M+H)⁺ | 567.12903 | 566.1216 | 0.57 | 96.92 |
| 25.304 | β-Eudesmol | C15H26O | (M+NH₄)⁺ | 240.23212 | 222.1983 | -0.16 | 99.62 |
| 27.499 | Glabrene | C20H18O4 | (M+H)⁺ | 323.12773 | 322.1205 | -0.06 | 98.67 |
| 28.086 | Cucurbitacin | C32H44O8 | (M+Na)⁺ | 579.29328 | 556.304 | 0.66 | 98.08 |
| 29.056 | Tangeretin | C20H20O7 | (M+H)⁺ | 373.12817 | 372.121 | 0.26 | 95.56 |
| 30.753 | Corylin | C20H16O4 | (M+H)⁺ | 321.11197 | 320.1048 | -0.35 | 99.53 |
| 30.909 | Celastrol | C29H38O4 | (M+Na)⁺ | 473.26548 | 450.2763 | -1.69 | 95.06 |
| 30.925 | Cinobufagin | C26H34O6 | (M+NH₄)⁺ | 460.26959 | 442.2358 | 0.58 | 99.16 |
| 31.703 | Alisol F | C30H48O5 | (M+Na)⁺ | 511.3392 | 488.3498 | -0.83 | 97.2 |
| 32.308 | Alpinumisoflavone | C20H16O5 | (M+H)⁺ | 337.10707 | 336.0998 | 0.21 | 98.74 |
| 32.741 | Nandrolone | C18H26O2 | (M+NH₄)⁺ | 292.22707 | 274.1933 | -0.12 | 98.88 |
| 34.541 | Simvastatin | C25H38O5 | (M+NH₄)⁺ | 436.30574 | 418.272 | 0.12 | 98.3 |
| 36.963 | Catharanthine | C21H24N2O2 | (M+H)⁺ | 337.19101 | 336.1839 | 0.25 | 98.09 |
| 40.93 | 10-Gingerol | C21H34O4 | (M+H)⁺ | 351.25274 | 350.2456 | -0.44 | 98.23 |
| 42.401 | Epibrassinolide | C28H48O6 | (M+NH₄)⁺ | 498.37868 | 480.3449 | -0.37 | 96.74 |
